# Effect of dietary β-mannanase supplementation on growth performance, intestinal morphology, digesta viscosity, and nutrient utilization in broiler chickens: Meta-analysis and meta-regression

**DOI:** 10.5713/ab.24.0459

**Published:** 2024-08-26

**Authors:** Hyun Woo Kim, Chan Ho Kwon, Ji Hye Lee, Min Sung Kang, Dong Yong Kil

**Affiliations:** 1 Department of Animal Science and Technology, Chung-Ang University, Anseong 17546, Korea

**Keywords:** β-Mannanase, Broiler Chicken, Growth Performance, Meta-analysis, Meta-regression

## Abstract

**Objective:**

The present study aimed to investigate the effectiveness of dietary β-mannanase supplementation on growth performance, intestinal morphology, digesta viscosity, and dietary nutrient utilization in broiler chickens through a meta-analysis. The effects were further examined by a meta-regression analysis with activity levels of β-mannanase in broiler diets.

**Methods:**

A total of 23 studies, which were conducted in 11 countries and completed between December 2003 and August 2023, were selected for this meta-analysis. The standardized mean difference and its 95% confidence interval were calculated as the effect size metrics using random effect model, with *I*^2^ value being utilized to measure heterogeneity. Investigated measurements included body weight gain (BWG), feed intake, feed conversion ratio (FCR), villus height (VH), crypt depth (CD), VH:CD ratio, digesta viscosity, nitrogen-corrected metabolizable energy (AME_n_), apparent ileal digestibility (AID), and apparent total tract retention (ATTR) of dry matter (DM), gross energy (GE), and nitrogen (N). All statistical analyses were performed using R version 4.3.3.

**Results:**

Results revealed significant positive effects of dietary β-mannanase supplementation on BWG (p = 0.005), FCR (p<0.001), VH (p<0.001), VH:CD (p<0.001), digesta viscosity (p<0.001), AME_n_ (p = 0.011), AID of GE (p = 0.002) and N (p = 0.003), and ATTR of DM (p = 0.019), GE (p = 0.002), and N (p = 0.005) in broiler chickens. In the meta-regression analysis, increasing activity levels of β-mannanase in broiler diets increased VH:CD (p< 0.001; R^2^ = 79.2%) and AID of N (p = 0.038; R^2^ = 67.4%).

**Conclusion:**

The current meta-analysis indicates that dietary β-mannanase supplementation improves energy and nutrient utilization in broiler diets possibly by decreasing digesta viscosity and enhancing intestinal morphology in broiler chickens. These beneficial effects can contribute to improved growth performance in broiler chickens.

## INTRODUCTION

Identifying factors that inhibit energy and nutrient utilization in animal diets, as well as developing potential solutions to mitigate the negative impact of the factors, are essential for optimizing animal production. The most critical anti-nutritional factor in diets for monogastric animals is considered non-starch polysaccharides (NSP), in particular for soluble NSP [1]. β-Mannan is a prevalent type of soluble NSP in plant protein ingredients, often found in relatively high amounts [2]. It has been demonstrated that high amounts of β-mannan in poultry diets have adverse impacts on productive performance and health by increased digesta viscosity, aggravation of intestinal health, and decreased energy and nutrient utilization in diets [3]. Consequently, to alleviate the anti-nutritional effects of β-mannan in poultry diets, dietary supplementation of β-mannanase as an exogenous enzyme is widely practiced in the poultry industry [3]. However, despite its purported potential actions, the results of dietary β-mannanase supplementation have been highly variable in previous broiler experiments.

Meta-analysis is a quantitative statistical approach that integrates reported data from various previous studies in order to draw comprehensive insights [4]. Recently, the meta-analysis is increasingly adopted across various fields of animal science to resolve conflicting findings from similar research topics [5]. Kiarie et al [6] conducted a meta-analysis for the effect of dietary β-mannanase supplementation on growth performance in broiler chickens, concluding a significant improvement in broiler performance. Similarly, the recent meta-analysis of Poulsen et al [7] also indicated overall changes in intestinal structure and functions in broiler chickens following dietary β-mannanase supplementation. However, there remains a lack of meta-analysis quantifying the effectiveness of dietary β-mannanase supplementation on intestinal structure, digesta viscosity, and dietary nutrient utilization, considering the potential modes of action of dietary β-mannanase in broiler chickens. Therefore, it is necessary to conduct the meta-analysis to integrate previous studies regarding the potential mode of actions to investigate its contribution to the improvement in broiler performance.

Therefore, the present study aimed to assess the effectiveness of dietary β-mannanase supplementation in broiler chickens with regard to growth performance, intestinal morphology, digesta viscosity, and dietary nutrient utilization through a meta-analysis. Furthermore, a meta-regression analysis was also conducted to investigate the effect of varying activity levels of β-mannanase in broiler diets.

## MATERIALS AND METHODS

### Inclusion and exclusion criteria

**Figure 1:**
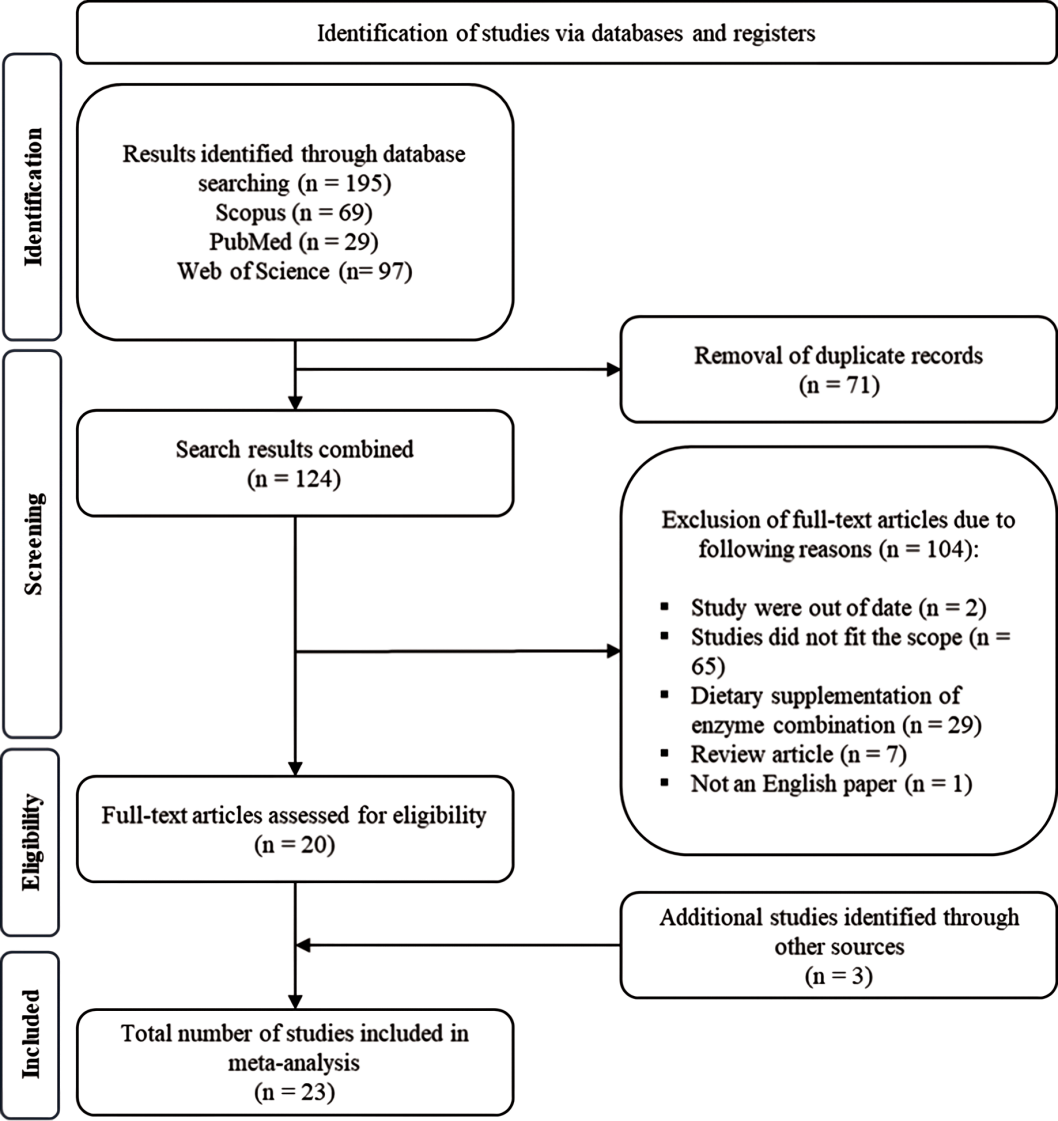
Selection and evaluation processes of published articles following the Preferred Reporting Items for Systemic Reviews and Meta-Analysis (PRISMA) protocol.

The process of search, selection, and evaluation of published articles for the present meta-analysis adhered to the Preferred Reporting Items for Systematic Reviews and Meta-Analysis (PRISMA) guidelines (Figure 1; [8]). A comprehensive search was conducted across multiple scientific databases including Scopus, PubMed, and Web of Science to identify the studies investigating the impact of dietary β-mannanase supplementation in broiler chickens. The keywords ‘broiler chickens’ and ‘β-mannanase’ were primarily utilized in this search. The trials included in the meta-analysis were selected based on specific eligibility criteria from an initial pool. These criteria included: i) Each study must include a control group with a standard basal diet and treatment groups with dietary β-mannanase supplementation; ii) The concentration and activity of β-mannanase utilized in each treatment diet must be clearly reported; iii) No enzyme other than β-mannanase should be supplemented to diets; iv) Comprehensive information describing the experimental factors must have been provided or made available upon the request from authors; v) Each study must report information pertaining to one or more of growth performance, intestinal morphology, digesta viscosity, and dietary energy and nutrient utilization. A total of 23 published studies met the inclusion criteria and were selected for the current meta-analysis. These selected studies were conducted across 11 countries and completed between December 2003 and August 2023.

### Data extraction

In accordance with the aforementioned inclusion and exclusion criteria, the considered study factors included the publication year, breeds of broiler chickens, replicates per treatment, study country, experimental period, and calculated or analyzed activity of β-mannanase in diets (Table 1). The investigated measurements included body weight gain (BWG), feed intake (FI), feed conversion ratio (FCR), villus height (VH), crypt depth (CD), VH:CD ratio, digesta viscosity, as well as nitrogen-corrected apparent metabolizable energy (AME_n_), apparent ileal digestibility (AID), and apparent total tract retention (ATTR) of dry matter (DM), gross energy (GE), and nitrogen (N) in diets.

The values for means, standard deviations (SD), and sample sizes for each measurement were collected for both the control group without dietary β-mannanase supplementation and the treatment group with dietary β-mannanase supplementation. In cases where the SD was not reported, the value was derived by multiplying the reported standard error (SE) values of the means by the square root of the sample size, as outlined by Higgins et al [9]. Moreover, unless individual SD or SE values were presented for each control and treatment group within the trials, a pooled SE or SD was used for both control and treatment group.

### Meta-analysis and meta-regression procedure

The standardized mean difference (SMD) and its 95% confidence interval (CI) were chosen as the effect size metrics for evaluating the responses of broiler chickens to dietary β-mannanase supplementation. The SMD represents the mean difference between the control group and β-mannanase treatment group, which was standardized by the SD of both groups [10]. The significance of effects of dietary β-mannanase supplementation on SMD was set at p<0.05. To access the variation in results among selected studies, the *I*^2^ statistics was adopted to estimate the heterogeneity [11]. In the current meta-analysis, all values for the *I*^2^ statistics were greater than 50% at the significance level of p<0.05, indicating the presence of significant heterogeneity in results among selected studies [12]. As a consequence, a random-effects model was employed for all the meta-analysis in this study [13]. A forest plot was generated to visualize the effects of dietary β-mannanase supplementation. Each study was depicted as an individual point on the forest plot, indicating the effect size, CI, and weighted contribution to each study, based on a random-effects model. Additionally, the publication bias in this meta-analysis was assessed graphically using a funnel plot [14].

A meta-regression was conducted to investigate the role of a specific covariate in the heterogeneity of treatment effects among studies. In the current study, total activity levels of β-mannanase supplemented in broiler diets was considered as the covariate. If the effect of the covariate was found to be significant, covariate bubble plots were generated to further analyze the meta-regression results [15]. All statistical analyses were performed using R version 4.3.3 [16]. The meta-analysis was conducted using the ‘meta’ package (version 7.0.0), whereas the meta-regression was performed using the ‘metafor’ package (version 4.6.0).

## RESULTS

### Literature retrieval and data extraction

A total of 195 studies were identified through a search of the public database and 71 duplicated studies were initially removed. The remaining 124 articles were thoroughly examined using the exclusion criteria, finally resulting in the selection of 20 studies. In addition, 3 more studies were found through other databases including Google Scholar. In total, 23 studies were finally included in the current meta-analysis (Figure 1). The detailed results of the data extraction and collation are presented in Table 1.

### Effects of dietary β-mannanase on growth performance

**Figure 2:**
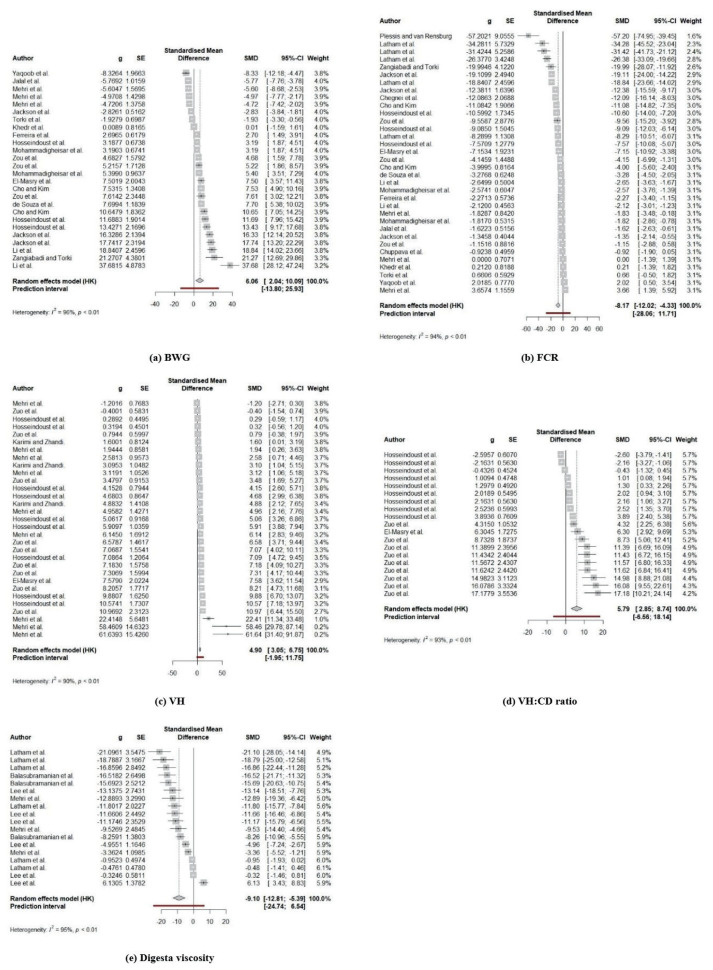
The forest plot for the results of dietary β-mannanase supplementation on growth performance and intestinal measurements in broiler chickens. (a) body weight gain (BWG), (b) feed conversion ratio (FCR), (c) villus height (VH), (d) VH to CD ratio (VH:CD), (e) digesta viscosity.

The effects of dietary β-mannanase supplementation on growth performance in broiler chickens were assessed by analyzing data from 26 trials for BWG, 29 trials for FI, and 34 trials for FCR from selected studies (Table 2). Due to considerable heterogeneity in BWG (*I*^2^ = 96.1%; p<0.001), FI (*I*^2^ = 94.7%; p<0.001), and FCR (*I*^2^ = 94.2%; p<0.001) among studies, a random-effects model was employed for this meta-analysis. The results revealed a significant positive effect of dietary β-mannanase supplementation on BWG (p = 0.005) and FCR (p<0.001) in broiler chickens. Moreover, the forest plot for BWG and FCR demonstrated that dietary β-mannanase supplementation led to an improvement in BWG (SMD = 6.06, 95% CI [2.04, 10.09]) and FCR (SMD = −8.17, 95% CI [−12.02, −4.33]) when comparing the control and treatment groups (Figure 2). However, no such an effect on FI was observed. As increasing activity levels of β-mannanase in broiler diets were hypothesized to affect the magnitude of changes in broiler performance, a meta-regression was performed with β-mannanase activity in diets as a covariate; however, significant effects were not detected (Table 3).

### Effects of dietary β-mannanase on intestinal morphology and digesta viscosity

**Figure 3:**
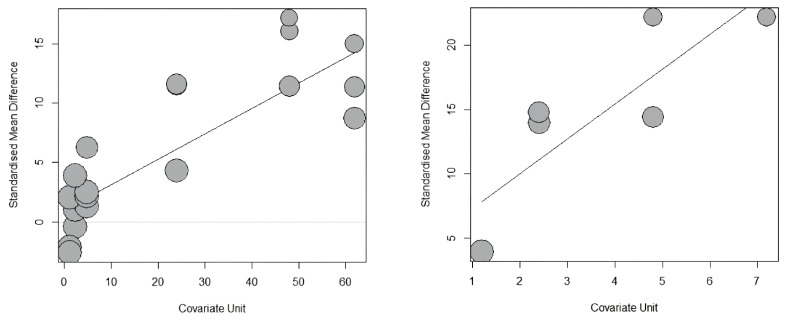
Meta-regression results of dietary β-mannanase supplementation on villus height and crypt depth ratio (VH:CD) and apparent ileal digestibility (AID) of nitrogen (N) in broiler chickens. The effect size (y-axis) is presented as standardized mean differences (SMD), whereas the covariate (x-axis) is presented as β-mannanase activity per g of diets (U/g).

The effect of dietary β-mannanase supplementation on intestinal morphology including VH, CD, VH:CD, and digesta viscosity in broiler chickens was explored using 31 trials, 31 trials, 19 trials, and 18 trials, respectively, from selected studies (Table 2). The random-effects model was also employed for this meta-analysis due to significant heterogeneity observed in VH (*I*^2^ = 90.3%; p<0.001), CD (*I*^2^ = 93.8%; p<0.001), VH:CD (*I*^2^ = 93.5%; p<0.001), and digesta viscosity (*I*^2^ = 94.7%; p<0.001) among studies. The meta-analysis demonstrated that dietary β-mannanase supplementation significantly increased VH (p<0.001; SMD = 4.90, 95% CI [3.05, 6.75]) and VH:CD (p<0.001; SMD = 5.79, 95% CI [2.85, 8.74]), but decreased digesta viscosity (p<0.001; SMD = −9.10, 95% CI [–12.81, −5.39]) in broiler chickens (Figure 2). However, no effect of dietary β-mannanase supplementation on CD was observed. In the meta-regression analysis, increasing activity levels of β-mannanase in diets led to a significant increase in VH:CD (p<0.001; R^2^ = 79.2%), whereas no effects on VH, CD, and digesta viscosity were observed (Table 3; Figure 3).

### Effects of dietary β-mannanase on energy and nutrient utilization

**Figure 4:**
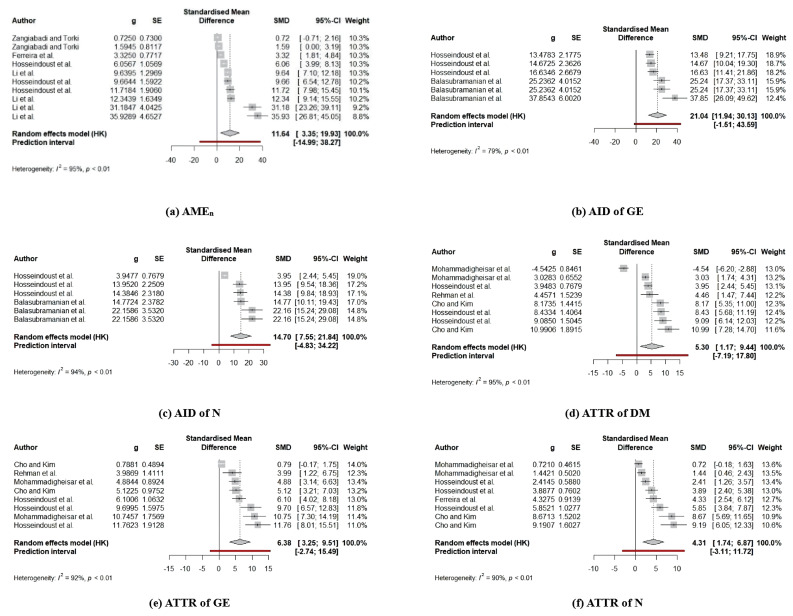
The forest plot for the results of dietary β-mannanase supplementation on energy and nutrient utilization in broiler chickens. (a) nitrogen-corrected apparent metabolizable energy (AME_n_), (b) apparent ileal digestibility (AID) of gross energy (GE), (c) AID of nitrogen (N), (d) apparent total tract retention (ATTR) of DM, (e) ATTR of GE, and (f) ATTR of N.

A total of 10 trials, 6 trials, and 8 trials from selected studies were analyzed to elucidate the effect of dietary β-mannanase supplementation on AME_n_, AID, and ATTR of energy and nutrients in broiler diets, respectively (Table 4). Significant heterogeneity was noted in AME_n_ (*I*^2^ = 95.4%; p<0.001), AID of DM (*I*^2^ = 94.6%; p<0.001), GE (*I*^2^ = 79.1%; p<0.001), and N (*I*^2^ = 93.9%; p<0.001), as well as ATTR of DM (*I*^2^ = 95.1%; p<0.001), GE (*I*^2^ = 92.4%; p<0.001), and N (*I*^2^ = 90.2%; p<0.001) among studies. Therefore, similar to the analysis conducted for growth performance and intestinal measurements, a random-effects model was utilized for the meta-analysis. The results represented a significant improvement in AME_n_ (p = 0.011; SMD = 11.64, 95% CI [3.35, 19.93]), AID of GE (p = 0.002; SMD = 21.04, 95% CI [11.94, 30.13]) and N (p = 0.003; SMD = 14.70, 95% CI [7.55, 21.84]), and ATTR of DM (p = 0.019; SMD = 5.30, 95% CI [1.17, 9.44]), GE (p = 0.002; SMD = 6.38, 95% CI [3.25, 9.51]), and N (p = 0.005; SMD = 4.31, 95% CI [1.74, 6.87]) in broiler diets by dietary β-mannanase supplementation (Figure 4). In the meta-regression analysis, increasing activity levels of β-mannanase in broiler diets improved AID of N (p = 0.038; R^2^ = 67.4%), whereas there were no effects of increasing activity levels of β-mannanase on AME_n_, AID of DM and GE, and ATTR of DM, GE, and N in broiler diets (Table 5; Figure 3).

### Publication bias

**Figure 5:**
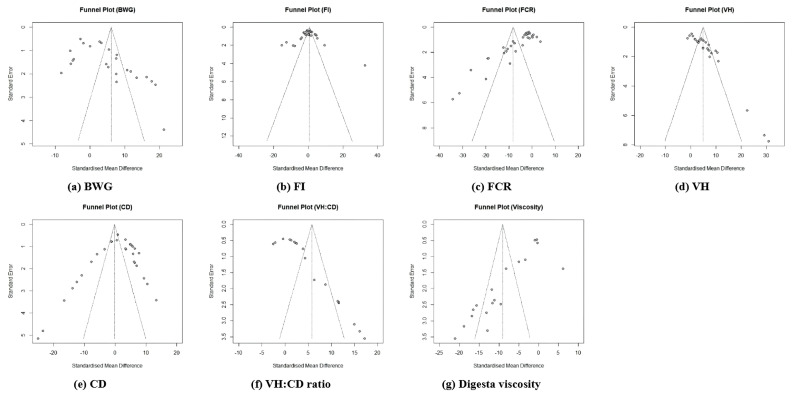
The funnel plot for the results of dietary β-mannanase supplementation on growth performance and intestinal measurements in broiler chickens. (a) body weight gain (BWG), (b) feed intake (FI), (c) feed conversion ratio (FCR), (d) villus height (VH), (e) crypt depth (CD), (f) VH to CD ratio (VH:CD), and (g) digesta viscosity.

**Figure 6:**
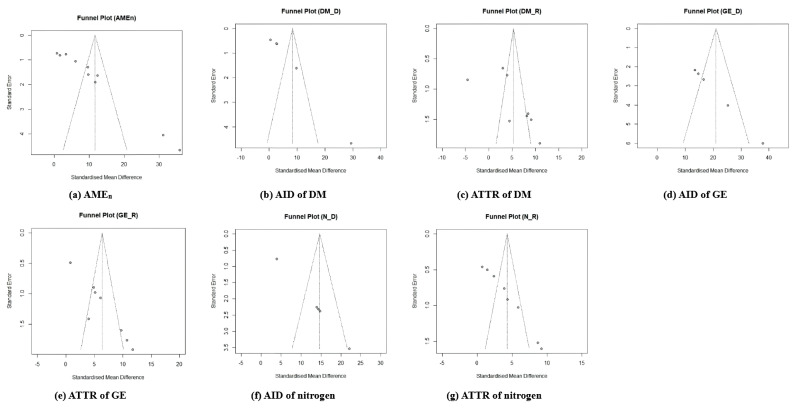
The funnel plot for the results of dietary β-mannanase supplementation on energy and nutrient utilization in broiler chickens. (a) nitrogen-corrected apparent metabolizable energy (AME_n_), (b) apparent ileal digestibility (AID) of dry matter (DM), (c) apparent total tract retention (ATTR) of DM, (d) AID of gross energy (GE), (e) ATTR of GE, (f) AID of nitrogen (N), and (g) ATTR of N.

The funnel plot was employed to assess the publication bias among selected studies regarding the effects of dietary β-mannanase supplementation in broiler chickens because it is known that high publication bias alters the shape of funnel plots in the meta-analysis. The current meta-analysis showed a lack of obvious symmetry in the measured funnel plots, as several trials were found outside the funnel plot (Figures 5, 6).

## DISCUSSION

β-Mannan is a linear polymer composed of the backbone of β-1,4-linked mannose, along with glucose and galactose residues [17]. β-mannan is recognized as one of the most significant soluble NSP in animal diets [18]. However, monogastric animals such as swine and poultry do not have the ability to produce endogenous digestive enzymes capable of breaking down dietary β-mannan, which exposes those animals to anti-nutritional effects of β-mannan. The adverse effects of dietary β-mannan are largely attributed to increased digesta viscosity in the gastrointestinal tract as characterized in other soluble NSP, resulting in decreased nutrient digestion and absorption [19,20]. Moreover, increased digesta viscosity is known to contribute to increased intestinal organ weight and unfavorable fermentation in the lower intestine [21]. Moreover, β-mannan has a molecular structure similar to certain pathogenic bacteria, which may induce unnecessary innate immune response [3]. Therefore, increasing use of energy and nutrient to boost immune responses may decrease energy and nutrient utilization towards growth performance [6]. Consequently, implementing proper dietary managements for either decreasing levels of or neutralizing β-mannan in poultry diets is imperative to safeguard poultry performance and health [22].

β-Mannanase is an exogenous enzyme supplemented in poultry diets to mitigate the adverse effects of dietary β-mannan [23]. However, the positive effect of dietary β-mannanase supplementation have been variable in previous studies due to considerable variations in experimental factors such as animals, diet compositions, feed ingredients, concentrations of dietary β-mannan, activity of β-mannanase in diets, and environmental conditions among studies. Therefore, to gain more comprehensive insights from highly variable research outcomes, the meta-analysis is increasingly adopted as a valuable tool in various fields of the animal studies [24].

In a recent study of Kiarie et al [6], a meta-analysis was conducted using 24 studies published between 2002 and 2019 to assess the effects of dietary β-mannanase supplementation, focusing particularly on broiler performance. The study showed significant improvements in both BWG and FCR in broiler chickens. Our meta-analysis also revealed significant effects on improving BWG and FCR in broiler chickens by feeding diets supplemented with β-mannanase although more recent broiler studies published from 2020 to 2023 were included in this study. Therefore, based on the previous and current meta-analysis, it can be concluded that dietary β-mannanase supplementation improves growth performance in broiler chickens despite evident variations in results among studies. Furthermore, considering that increasing activity of β-mannanase in diets may lead to a greater degradation of β-mannan in diets, the meta-regression analysis was performed using the activity levels of β-mannanase in diets as a covariate. However, no significant associations were identified between the activity levels of β-mannanase in diets and improvements in broiler performance. The reason for this lack of significance is unclear; however, it may be attributed to variations in animals and diet compositions, including differences in dietary β-mannan concentrations, among previous studies. Considering that most of the studies were conducted by supplementing more than the levels of β-mannanase recommended by suppliers (i.e., recommended level of Hemicell = 29 U/g; recommended level of CTCzyme = 0.4 U/g), it can be suggested that over-dosing of β-mannanase than recommended levels has little additional benefits on broiler performance.

Improvements in broiler performance by feeding diets supplemented with β-mannanase have been primarily associated with reduced digesta viscosity and enhanced intestinal morphology. These changes in intestinal characteristics contribute to improved energy and nutrient utilization in diets [25]. Consequently, we conducted a meta-analysis for intestinal measurements and dietary nutrient utilization in broiler chickens as affected by dietary β-mannanase supplementation.

Many previous studies measured digesta viscosity in broiler chickens to support the positive effect of dietary β-mannanase supplementation by promoting break down of viscous β-mannan in the gastrointestinal tract [26]. The present meta-analysis confirms a significant decrease in digesta viscosity by feeding diets supplemented with β-mannanase to broiler chickens. However, 4 trials out of 18 trials used in this meta-analysis reported no beneficial effects on digesta viscosity. It is appreciated that decreased digesta viscosity may have a positive impact on intestinal morphology with an increase in VH and VH:CD. This observation is related to the fact that highly viscous digesta promotes the slough-off of intestinal cells, resulting in decreased VH:CD with reducing VH but increasing CD [27]. Furthermore, dietary supplementation of β-mannanase decreased β-mannan-induced immune stimulations in the gastrointestinal tract by breaking down β-mannan, which may allow more energy and nutrients to be utilized for intestinal development [28]. The present meta-analysis also showed a significant increase in VH and VH:CD without affecting CD. Interestingly, our meta-regression analysis indicated a significant interaction between β-mannanase activity in broiler diets and VH:CD, suggesting that increasing activity levels of β-mannanase in diets may linearly improve VH:CD in the gastrointestinal tract of broiler chickens. The possible reason for this association is difficult to explain because of no interactive effects on other intestinal measurements including digesta viscosity, VH, and CD. However, it should be noted that only one study of Zuo et al [29] examined the effect of very high activity levels of β-mannanase in broiler diets, reporting a remarkable increase in VH:CD, such that its impact was possibly over-valued in this meta-regression analysis. It appears that removal of the data from Zuo et al [29] in the meta-regression analysis may result in no significant interaction between β-mannanase activity in diets and VH:CD. However, more research regarding the effect of varying activity levels of β-mannanase in broiler diets on intestinal measurements is required to validate the findings of our meta-regression analysis.

Improved intestinal morphology as well as decreased digesta viscosity by dietary β-mannanase supplementation as outlines in the current meta-analysis can contribute to increased energy and nutrient utilization in broiler diets because decreased digesta viscosity increases nutrient digestions by increasing access of digestive enzymes to macromolecules in the digesta [30]. Moreover, increased VH:CD extends the surface area of villi for promoting efficient nutrient absorptions [31]. For these reasons, the current meta-analysis also provides strong evidence for positive impact of dietary β-mannanase supplementation on energy and nutrient utilization (AID of GE and N, ATTR of DM, GE, and N) and further AME_n_ values for broiler diets. These findings are consistent with the previous meta-analysis reported improved AME_n_ values in broiler diets by dietary β-mannanase supplementation [6]. In the present study, despite the relatively smaller number of trials analyzed for energy and nutrient utilization than for growth performance and intestinal measurements, the effectiveness of dietary β-mannanase supplementation was more considerable. Thus, these results may suggest that improved broiler performance by dietary β-mannanase supplementation is caused by improving energy and nutrient utilization in diets. Moreover, with regard to measuring dietary energy and nutrient utilization in this study, the meta-regression analysis revealed a linear increase in the AID of N as activity levels of β-mannanase in diets were increased. However, no such an effect was identified in other measurements. Previous studies have reported a linear increase in protein digestibility by increasing activity levels of β-mannanase in diets fed to pigs [32] and poultry [28], which supports our results of the meta-regression. However, it still remains unknown why increasing activity levels of β-mannanase were found to induce a linear improvement solely in the AID of N in broiler diets. Very limited number of studies investigating the relationship between dietary β-mannanase activity and nutrient utilization in broiler diets restricts our further explanation.

The current meta-analysis indicates significant heterogeneity in all measurements. The primary reason for this heterogeneity can be attributed to highly variable experimental conditions such as animals, environment, and experimental design among studies [33]. Therefore, a meta-regression is often proposed to identify specific factors affecting the heterogeneity and further to investigate the relationship between those factors and the research outcomes [15]. In this study, we performed the meta-regression with β-mannanase activity in broiler diets as a covariate, based on the hypothesis that increasing activity levels of β-mannanase in broiler diets may influence the effectiveness of dietary β-mannanase supplementation on growth performance, intestinal measurements, and utilization of energy and nutrients in diets. However, we failed to find the significant interactions for most measurements, except VH:CD and AID of N. This result may suggest that increasing activity levels of β-mannanase in diets may have little impact on broiler chickens. The reason for this result may be associated with the fact that dietary β-mannanase activity used in most previous studies is very close to or exceeds the recommended levels provided by the manufacturer. Therefore, it is unlikely that the activity levels of β-mannanase in broiler diets is the main cause of high heterogeneity observed in this meta-analysis. Consequently, it is suggested that other factors such as feed particle size, dietary β-mannan concentration, enzyme stability, and experimental condition may contribute to high heterogeneity. Limited studies have been conducted regarding possible factors affecting the efficacy of dietary β-mannanase. Therefore, further studies are necessary to identify the specific reasons for high heterogeneity of effects of dietary β-mannanase supplementation in broiler chickens. Furthermore, it is important to note that there may be a possible publication bias, as there is a tendency to favor publication of studies with significant results [5], which may lead to the inclusion of only positive results in the meta-analysis, potentially influencing the heterogeneity among studies [34].

## CONCLUSION

The present meta-analysis indicates that dietary β-mannanase supplementation improves energy and nutrient utilization in broiler diets by decreasing digesta viscosity and enhancing intestinal morphology in broiler chickens. These beneficial effects are likely to contribute to improved growth performance in broiler chickens. However, it should be acknowledged that the present study has a limitation due to high heterogeneity among research findings. Therefore, future meta-analysis should incorporate more research on the effects of dietary β-mannanase possibly with varying activity levels in broiler diets to improve the accuracy of the meta-analysis and meta-regression results.

## Figures and Tables

**Table 1:** Results of literature data extraction and collation regarding effect of dietary β-mannanase supplementation on growth performance, intestinal measurements, and dietary energy and nutrient utilization in broiler chickens

Author	Year	Breed	Replicate	Study country	Study period (d)	Activity of β-mannanase per gram of diets (U/g)	Measurements	Reference
de Souza et al	2023	Ross 308	12	Brazil	0–35	52.8	BWG, FI, FCR	[35]
Jalal et al	2023	Indian River	10	Jordan	0–35	80	BWG, FI, FCR	[36]
Chuppava et al	2022	Ross 308	9	Germany	7–33	171.1	FCR	[37]
Yaqoob et al	2022	Arbor Acres	5	Pakistan	8–35	32	BWG, FI, FCR	[38]
Mohammadigheisar et al	2021	Ross 308	10	Korea	1–35, 32–35	0.4	BWG, FI, FCR, ATTR of DM, GE, and N	[39]
Hosseindoust et al	2019	Ross 308	10	Korea	1–35, 33–35	1.2, 2.4, 4.8	BWG, FI, FCR, VH, CD, VH:CD AME_n_, AID of DM, GE, and N, ATTR of DM, GE, and N	[25]
Balasubramanian et al	2018	Ross 308	10	Korea	1–35	2.4, 4.8, 7.2	Viscosity, AID of DM, GE, and N	[40]
Latham et al	2018	Not specified	9	USA	0–42	24, 48	FCR, Viscosity, AID of GE	[41]
El-Masry et al	2017	Ross 308	4	Egypt	0–37	4.8	BWG, FI, FCR, VH, CD, VH:CD	[42]
Ferreira et al	2016	Cobb 500	8	Brazil	13–21	0.4	AME_n_, ATTR of N	[43]
Rehman et al	2016	Hubbard	3	Pakistan	36–42	48	FI, ATTR of DM and GE	[44]
Karimi and Zhandi	2015	Ross 308	4	Iran	21	140	VH, CD, VH:CD	[45]
du Plessis and van Rensburg	2014	Ross 308	10	South Africa	0–35	159.5	FI, FCR	[46]
Zuo et al	2014	Yellow-feathered broiler	6	China	21	24, 48, 72	VH, CD, VH:CD	[29]
Cho and Kim	2013	Ross 308	9	Korea	0–28, 21	0.04	BWG, FI, FCR, ATTR of DM, GE, and N	[47]
Chengeni et al	2011	Cobb 500	9	USA	0–49	66	FI, FCR	[48]
Torki	2011	Cobb 500	6	Iran	0–49	66	BWG, FI, FCR	[49]
Li et al	2010	Arbor Acres	15	China	0–42, 21, 42	10, 20	FI, BWG, FCR, AME_n_	[28]
Mehri et al	2010	Cobb 500	4	Iran	0–42	82.5, 115.5, 148.5	BWG, FI, FCR, VH, CD, Viscosity	[50]
Zangiabadi and Torki	2010	Arbor Acer	4	Iran	21–28	64	BWG, FI, FCR, AME_n_	[51]
Zou et al	2006	Avine	3	China	0–42	41.3, 82.5, 123.8	BWG, FI, FCR	[52]
Jackson et al	2004	Cobb 500	15	Spain	0–42	50, 80, 110	BWG, FI, FCR	[26]
Lee et al	2003	Cobb	6	USA	21, 42	109, 436	Viscosity	[19]

BWG, body weight gain; FI, feed intake; FCR, feed conversion ratio; ATTR, apparent total tract retention; DM, dry matter; GE, gross energy; N, nitrogen; AME_n_, nitrogen-corrected apparent metabolizable energy; AID, apparent ileal digestibility; VH, villus height; CD, crypt depth; VH:CD, villus height and crypt depth ratio.

**Table 2:** Meta-analysis results of dietary β-mannanase supplementation on growth performance and intestinal measurements in broiler chickens

Indexes		Trials	Effect size estimates			Heterogeneity test	
	
			SMD	CI (95%)	p-value	*I*^2^-value (%)	p-value
Growth performance	BWG	26	6.06	[2.04, 10.09]	0.005	96.1	<0.001
	FI	29	0.71	[−3.98, 5.41]	0.758	94.7	<0.001
	FCR	34	−8.17	[−12.02, −4.33]	<0.001	94.2	<0.001
Intestinal morphology	VH	31	4.90	[3.05, 6.75]	<0.001	90.3	<0.001
	CD	31	−2.06	[−3.55, 3.14]	0.901	93.8	<0.001
	VH:CD	19	5.79	[2.85, 8.74]	<0.001	93.5	<0.001
Digesta viscosity		18	−9.10	[−12.81, −5.39]	<0.001	94.7	<0.001

SMD, standardized mean difference; CI, confidence interval; BWG, body weight gain; FI, feed intake; FCR, feed conversion ratio; VH, villus height; CD, crypt depth; VH:CD, VH to CD ratio.

**Table 3:** Meta-regression results of dietary β-mannanase supplementation with activity levels of β-mannanase as a covariate on growth performance and intestinal measurements in broiler chickens

Indexes		Trials	Test of units			Test of residual variance (intercept)			R^2^ (%)
	
			Coefficient	CI (95%)	p-value	Coefficient	CI (95%)	p-value	
Growth performance	BWG	26	−0.08	[−0.17, 0.01]	0.069	9.77	[4.19, 15.36]	0.001	10.1
	FI	29	0.09	[−0.01, 0.19]	0.088	−3.55	[−10.30, 3.20]	0.290	0.0
	FCR	34	−0.01	[−0.08, 0.08]	0.940	−8.07	[−13.55, −2.60]	0.005	0.0
Intestinal morphology	VH	31	−0.01	[−0.04, 0.03]	0.671	5.31	[2.58, 8.05]	<0.001	1.2
	CD	31	0.02	[−0.04, 0.08]	0.432	−1.67	[−6.72, 3.37]	0.503	0.0
	VH:CD	19	0.21	[0.14, 0.29]	<0.001	0.99	[−0.97, 2.95]	0.300	79.2
Digesta viscosity		18	0.01	[−0.01, 0.04]	0.298	−10.68	[−15.53, −5.83]	<0.001	0.7

CI, confidence interval; BWG, body weight gain; FI, feed intake; FCR, feed conversion ratio; VH, villus height; CD, crypt depth; VH:CD, VH to CD ratio.

**Table 4:** Meta-analysis results of dietary β-mannanase supplementation on energy and nutrient utilization in broiler diets

Indexes		Trials	Effect size estimates			Heterogeneity test	
	
			SMD	CI (95%)	p-value	*I*^2^-value (%)	p-value
AME_n_		10	11.64	[3.35, 19.93]	0.011	95.4	<0.001
AID	DM	6	8.41	[−2.11, 18.94]	0.095	94.6	<0.001
	GE	6	21.04	[11.94, 30.13]	0.002	79.1	<0.001
	N	6	14.70	[7.55, 21.84]	0.003	93.9	<0.001
ATTR	DM	8	5.30	[1.17, 9.44]	0.019	95.1	<0.001
	GE	8	6.38	[3.25, 9.51]	0.002	92.4	<0.001
	N	8	4.31	[1.74, 6.87]	0.005	90.2	<0.001

SMD, standardized mean difference; CI, confidence interval; AME_n_, nitrogen-corrected apparent metabolizable energy; AID, apparent ileal digestibility; ATTR, apparent total tract retention; DM, dry matter; GE; gross energy; N, nitrogen.

**Table 5:** Meta-regression results of dietary β-mannanase supplementation with activity levels of β-mannanase as a covariate on energy and nutrient utilization in broiler diets

Indexes		Trials	Test of units			Test of residual variance (intercept)			R^2^ (%)
	
			Coefficient	CI (95%)	p-value	Coefficient	CI (95%)	p-value	
AME_n_		10	−0.11	[−0.49, 0.26]	0.499	13.93	[2.53, 25.32]	0.023	0.0
AID	DM	6	2.74	[−4.50, 10.48]	0.381	0.94	[−23.18, 25.05]	0.919	0.0
	GE	6	2.75	[−1.43, 6.95]	0.142	10.93	[−5.89, 27.75]	0.146	32.9
	N	6	2.70	[0.24, 5.16]	0.038	4.63	[−5.11, 14.37]	0.257	67.4
ATTR	DM	8	−0.12	[−0.31, 0.29]	0.927	5.41	[0.34, 10.48]	0.040	0.0
	GE	8	−0.04	[−0.27, 0.18]	0.647	6.72	[2.95, 10.48]	0.005	0.0
	N	8	−0.19	[−2.04, 1.66]	0.812	4.58	[0.90, 8.26]	0.023	0.0

CI, confidence interval; AME_n_, nitrogen-corrected apparent metabolizable energy; AID, apparent ileal digestibility; ATTR, apparent total tract retention; DM, dry matter; GE; gross energy, N; nitrogen.
